# DNA methylation at *IL32* in juvenile idiopathic arthritis

**DOI:** 10.1038/srep11063

**Published:** 2015-06-09

**Authors:** Braydon Meyer, Raul A. Chavez, Jane E. Munro, Rachel C. Chiaroni-Clarke, Jonathan D. Akikusa, Roger C. Allen, Jeffrey M. Craig, Anne-Louise Ponsonby, Richard Saffery, Justine A. Ellis

**Affiliations:** 1 Genes, Environment & Complex Disease, Murdoch Childrens Research Institute, Parkville, Victoria, Australia; 2 Department of Paediatrics, University of Melbourne, Victoria, Australia; 3 Arthritis & Rheumatology, Murdoch Childrens Research Institute, Parkville, Victoria, Australia; 4 Paediatric Rheumatology Unit, Royal Children’s Hospital, Parkville, Victoria, Australia; 5 Environmental & Genetic Epidemiology Research, Murdoch Childrens Research Institute, Parkville, Victoria, Australia; 6 Cancer & Disease Epigenetics, Murdoch Childrens Research Institute, Parkville, Victoria, Australia

## Abstract

Juvenile idiopathic arthritis (JIA) is the most common autoimmune rheumatic disease of childhood. We recently showed that DNA methylation at the gene encoding the pro-inflammatory cytokine interleukin-32 (*IL32*) is reduced in JIA CD4+ T cells. To extend this finding, we measured *IL32* methylation in CD4+ T-cells from an additional sample of JIA cases and age- and sex-matched controls, and found a reduction in methylation associated with JIA consistent with the prior data (combined case-control dataset: 25.0% vs 37.7%, p = 0.0045). Further, JIA was associated with reduced *IL32* methylation in CD8+ T cells (15.2% vs 25.5%, p = 0.034), suggesting disease-associated changes to a T cell precursor. Additionally, we measured regional SNPs, along with CD4+ T cell expression of total *IL32*, and the γ and β isoforms. Several SNPs were associated with methylation. Two SNPs were also associated with JIA, and we found evidence of interaction such that methylation was only associated with JIA in minor allele carriers (e.g. rs10431961 p_interaction_ = 0.011). Methylation at one measured CpG was inversely correlated with total *IL32* expression (Spearman r = −0.73, p = 0.0009), but this was not a JIA-associated CpG. Overall, our data further confirms that reduced *IL32* methylation is associated with JIA, and that SNPs play an interactive role.

Juvenile idiopathic arthritis (JIA) is the most common autoimmune rheumatic disease in children, affecting approximately one per thousand of European descent, and causing significant disability^1^,^2^. JIA is considered to be a complex disease, and as such, risk is presumed to be determined by both genetic and environmental factors^3^. Recent work has identified a number of genetic risk loci, including HLA, *PTPN22*, and genes of the IL-2 pathway^4^,^5^. Specific environmental factors potentially contributing to risk remain largely uncharacterised; however, a role for environmental exposures is supported by numerous lines of evidence for other autoimmune diseases^6^.

Epigenetic modification, including DNA methylation, is widely believed to provide a mechanism through which environmental and genetic risk factors interact to promote disease^7^. Indeed, there is a large and rapidly-growing body of evidence for altered DNA methylation in individuals with various autoimmune diseases, including rheumatoid arthritis (RA)^8^,^9^,^10^,^11^, systemic lupus erythematosus^12^,^13^,^14^, inflammatory bowel disease^15^,^16^,^17^ and type 1 diabetes^18^,^19^. We recently performed a pilot genome-scale analysis of peripheral blood CD4+ T cell DNA methylation in (predominantly oligoarticular course) JIA^20^. Among the genomic regions showing evidence of differential methylation in JIA cases relative to age- and sex-matched controls, was the regulatory region of the cytokine interleukin-32 gene (*IL32)*. IL-32 promotes the production of a number of pro-inflammatory molecules, including tumour necrosis factor alpha (TNFα) and interleukin 6^21^,^22^,^23^, cytokines that are targeted in biologic therapies for JIA^24^, and is therefore a biologically plausible candidate gene for JIA pathogenesis.

IL-32 has been implicated in a number of different biological pathways, including cell death, host defence, and immune function^25^,^26^. Several lines of evidence also suggest that IL-32 plays an important role in inflammatory arthritis (for review, see^27^). Shoda *et al*. showed that *IL32* expression is prominent in synovial-infiltrated lymphocytes of RA patients, and overexpression has the capacity to exacerbate inflammatory arthritis in mice^28^. Park *et al*. showed that injection of IL-32 into mouse knee joints led to increased pro-inflammatory cytokine production, synovial inflammation and bone destruction^29^. Promotion of joint inflammation by IL-32 appears to be TNFα dependent, since IL-32 did not induce inflammation in TNFα-deficient mice^30^.

Six isoforms of IL-32 exist (γ, β, α, δ, ε, ζ), as the result of mRNA splicing^21^,^31^,^32^. Heinhuis *et al*. observed that IL-32γ expression was significantly greater in RA compared to osteoarthritis (OA) synovial biopsies, and that in RA synovial tissues, IL-32γ expression was correlated with expression of pro-inflammatory cytokines including TNFα^33^. Splicing of IL-32γ to IL-32β reduced pro-inflammatory potency, demonstrated by a lack of correlation of IL-32β with TNFα or IL-6, suggesting that splicing may serve as a mechanism to control inflammation^33^.

Given our prior evidence of reduced DNA methylation at *IL32* in CD4+ T cells of individuals with JIA, and mounting evidence for a role for IL-32 in inflammatory arthritis, we sought to generate further evidence that *IL32* plays a role in JIA. We investigated *IL32* DNA methylation in circulating CD4+ T cells in a further JIA case-control sample, and in an additional immune cell subset, CD8+ T cells, considered the impact of single nucleotide polymorphisms (SNPs) on DNA methylation, and explored the relationship between CD4+ T cell *IL32* methylation and *IL32* expression.

## Results

### A 5’ region of *IL32* is differentially methylated in JIA CD4+ T cells

**Figure 1:**
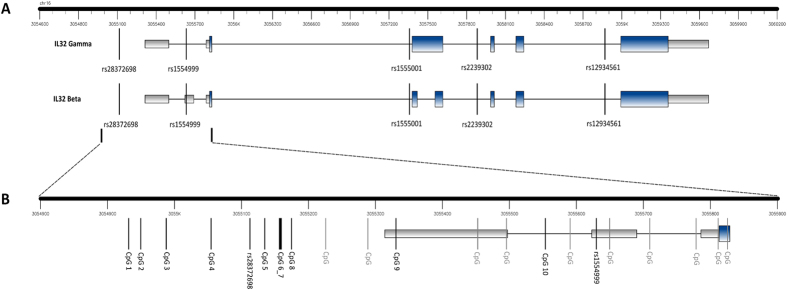
Graphical representation of the gene structure of *IL32*, and location of CpGs and SNPs measured in this study. A. *IL32* Gamma and Beta isoforms with regional SNPs annotated. B. Close-up of 5’ gene region with measured CpGs annotated, other unmeasured CpGs in the region are shown in grey. Blue bars = exons. Grey bars = UTRs. Hg18 co-ordinates.

**Figure 2:**
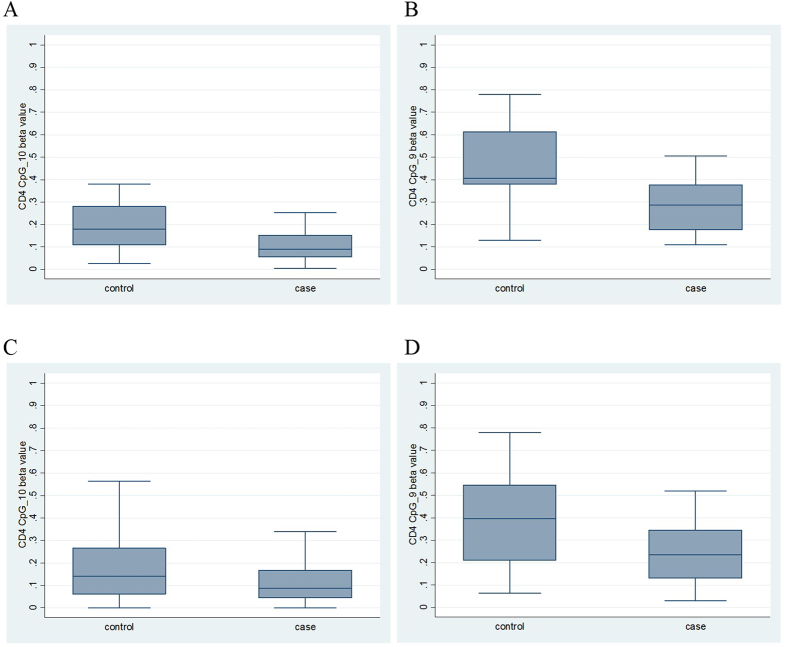
Methylation beta values in JIA cases and controls at the two significantly different *IL32* CpG sites. A: CpG_10 in replication samples. B: CpG_9 in replication samples. C: CpG_10 in original and replication samples combined. D: CpG_9 in original and replication samples combined.

We previously found evidence for differential methylation of two CpG sites (designated CpG_10 and CpG_9; Fig. 1) in the 5’ region of *IL32* in CD4+ T cells of children with JIA^20^. To confirm this association, we measured DNA methylation in the same genomic region (total of 10 CpG sites within nine assay units) in purified CD4+ T cells from a replication sample of 12 oligoarticular JIA cases and age- and sex-matched controls. The CD4+ *IL32* methylation beta values for each replication sample at each measured CpG unit are given in Supplementary Table 5. As with our previous pilot data, we identified a significant reduction in mean methylation in JIA cases relative to controls (CpG_10: 10.3% (95% CI 5.8–14.9%) vs 19.0% (95% CI 12.0–26.1%), t-test p = 0.032; CpG_9: 29.2% (95% CI 20.0–38.4%) vs 45.9% (95% CI 33.6–58.3%), t-test p = 0.028) (Fig. 2A,B). To maximise sample size for subsequent analyses, we combined all ‘original’ and ‘replication’ sample methylation data (Fig. 2C,D). Overall, mean DNA methylation in JIA cases at both CpG_10 and CpG_9 was significantly reduced relative to controls in the combined dataset (CpG_10: 10.7% (95% CI 7.9–13.7%) vs 17.1% (95% CI 12.3–21.9%), t test p = 0.025; CpG_9: 25.0% (95% CI 19.6–30.3%) vs 37.7% (95% CI 30.6–44.8%), p = 0.0045). Logistic regression demonstrated that, for CpG_9, a 1% increase in methylation was associated with a 5% decrease in the risk of JIA (OR = 0.95, 95% CI 0.92, 0.99, p = 0.008). CD4+ *IL32* methylation levels at seven other analytic units (comprising 8 CpG sites) were not associated with JIA (Supplementary Table 5).

To maximise homogeneity amongst the cases, we reanalysed the association of JIA with *IL32* methylation after removing the four polyarticular JIA cases from the ‘original’ sample set. Patterns of association were unchanged (data not shown).

### A similar pattern of *IL32* differential methylation is seen in JIA CD8+ T cells

In order to explore the potential cell specificity of the observed CD4+ T cell differential methylation in JIA, we measured *IL32* methylation in CD8+ T cells isolated simultaneously with the CD4+ T cells in the ‘replication’ case-control pairs. Resulting methylation values are given in Supplementary Table 5. For all CpGs measured, mean CD8+ T cell *IL32* methylation was lower than in matched CD4+ T cells. As with CD4+ T cells, CD8+ T cell methylation at CpG_10 and CpG_9 was associated with JIA (CpG_10: 5.9% (95% CI 2.5–9.3%) vs 11.6% (95% CI 7.1–16.2%), t-test p = 0.037; CpG_9: 15.2% (95% CI 9.1–21.4%) vs 25.5% (95% CI 17.7–33.3%), t-test p = 0.034). Logistic regression demonstrated that, for CpG_9, a 1% increase in methylation was marginally associated with a 10% decrease in the risk of being a JIA case (OR = 0.91, 95% CI 0.83, 1.00, p = 0.054). CD8+ *IL32* methylation at other measured CpGs was not associated with JIA.

We measured the correlation of CpG methylation within and between CD4+ and CD8+ T cells. In general, there was a high level of correlation between CpG sites within each cell type (Supplementary Tables 6 and 7). CpG_9 and CpG_10 were highly correlated in both CD4+ T cells (Pearson r = 0.86, p < 0.0001) and CD8+ T cells (Pearson r = 0.84, p < 0.0001). When methylation levels were compared between cell types, CpG_1, CpG_3, CpG_8, CpG_9 and CpG_10 in CD4+ T cells were significantly correlated with their equivalent CpG in CD8+ T cells (Table 1).

### SNPs in the *IL32* gene region are associated with methylation

**Figure 3:**
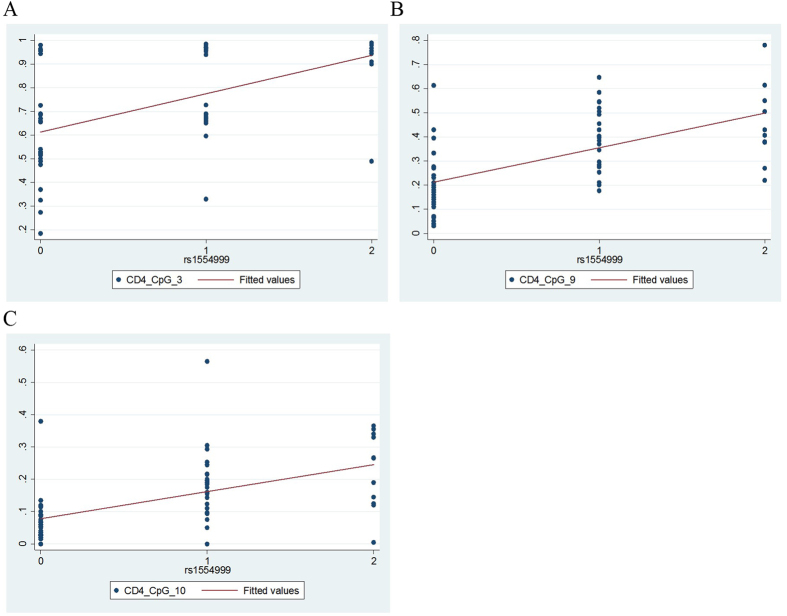
Correlations between genotype at rs1554999 and *IL32* β methylation values for CD4+ T cell CpGs (cases and controls combined). Only statistically significant correlations are shown. A. CD4 CpG_3, Pearson r = 0.54. B. CD4 CpG_9, Pearson r = 0.60. C. CD4 CpG_10, Pearson r = 0.54. SNP rs1554999 genotype 0 = CC, 1 = CA, 2 = AA (minor allele homozygote).

We found the genotypes of a number of SNPs in the *IL32* region of chromosome 16 to be correlated with methylation at specific CpG sites. Table 2 shows the nine SNPs that were significantly associated with at least one CpG methylation level in at least one cell type. Methylation at CpG_10 and CpG_9 in CD4+ T cells appeared to be most commonly impacted by genotype, associated with six SNPs and four SNPs respectively. The SNP with the strongest overall evidence for association with *IL32* methylation (high number of CpGs and low p value for association) was rs1554999. Figure 3 shows the correlation plots of rs1554999 with methylation at the three CpGs with which it is correlated in CD4+ T cells.

### CD4+ T cell *IL32* methylation remains associated with JIA after taking into account the effect of SNPs

We noted that two SNPs found to be associated with *IL32* methylation, rs10431961 and rs7188573, were also associated with JIA by logistic regression (rs10431961: OR = 2.74; 95% CI 1.23, 6.12; p = 0.014; rs7188573: OR = 3.14; 95% CI 1.32, 7.49, p = 0.010). Given this, we considered them as confounders in the logistic regression model assessing the association between methylation and JIA. After adjusting for these two SNPs, CD4+ T cell CpG_10 methylation no longer remained associated with JIA (OR = 0.96, 95% CI 0.91, 1.01, p = 0.11). However, the association was still apparent for CpG_9 (OR = 0.96, 95% CI 0.92, 0.99, p = 0.034). Again, these patterns of association were not materially altered by the removal of polyarticular JIA cases.

### Evidence of interaction between genotype and *IL32* methylation in JIA risk

We looked for evidence of interaction between SNPs rs10431961 and rs7188573 (main effect associations with JIA), and CD4+ DNA methylation at CpG_9. For rs10431961, we found that the association between JIA and CpG_9 methylation was present only in those with at least one minor allele (major allele homozygote group: AOR = 0.99, 95% CI 0.94–1.04, p = 0.71; heterozygote plus minor allele homozygote group: AOR = 0.85, 95% CI 0.74–0.98, p = 0.023). There was statistically significant evidence of multiplicative interaction between CpG_9 and rs10431961 (AOR_int_ = 0.86, 95% CI 0.76–0.97, p_int_ = 0.011). Similarly for rs7188573, we found that the association between JIA and CpG_9 was present only in those with at least one minor allele (major allele homozygote group: AOR = 0.98, 95% CI 0.93–1.03, p = 0.34; heterozygote plus minor allele homozygote group: AOR = 0.90, 95% CI 0.82–0.99, p = 0.028). Again, the test for interaction was statistically significant (AOR_int_ = 0.89, 95% CI 0.80–0.99, p_int_ = 0.039). SNPs rs10431961 and rs7188573 are in moderate linkage disequilibrium in our sample (r^2^ = 0.76). However, conditioning the above interaction analyses on the opposite SNP did not materially alter the outcomes, suggesting that these two SNPs may be interacting independently with CD4+ T cell DNA methylation at CpG_9. The outcomes of these analyses were not altered by removal of polyarticular JIA cases.

### Relationship between *IL32* methylation and expression in CD4+ T cells

**Figure 4:**
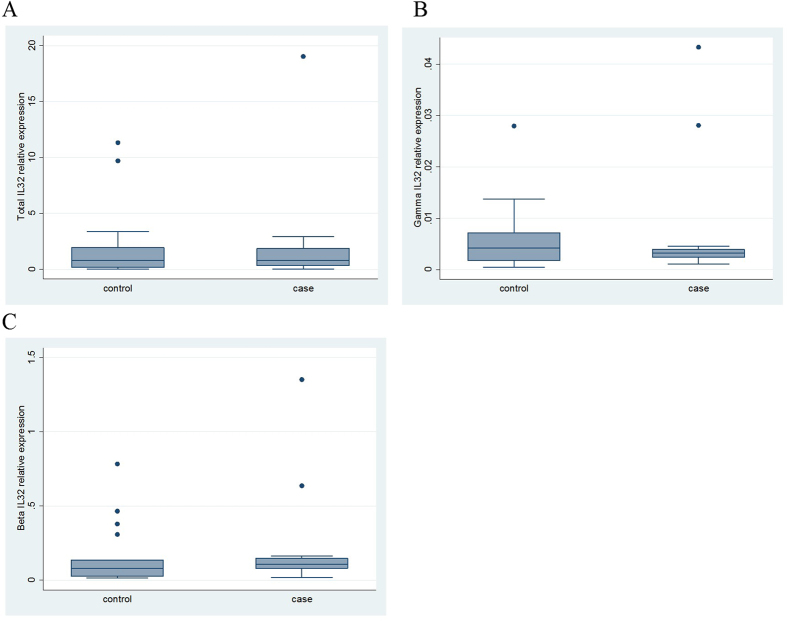
Distribution of relative expression of A. total *IL32*, B. *IL32*γ and C. *IL32*β in CD4+ T cells from cases and controls. *IL32* expression relative to *B2M* housekeeper gene expression.

In order to directly ascertain the functional relevance of the observed differential methylation of *IL32* in JIA, we measured expression of total *IL32* (the sum of all transcript variants), *IL32*γ, and *IL32*β in CD4+ T cells from JIA cases and controls. Expression values were normalised to the housekeeping gene *B2M*; normalisation to an alternative housekeeping gene, *RPLPO*, did not materially alter the values (data not shown). Figure 4 shows the distribution of relative expression values in JIA cases and controls. Supplementary Table 8 provides individual-level relative expression values. There was a large range of values for each measure of *IL32* expression in both cases and controls. Overall, we detected higher levels of expression of *IL32*β than *IL32*γ, consistent with prior evidence that *IL32*β is the more predominant *IL32* gene product in T cells^31^. Regression analysis adjusting for age, sex, and blood time to processing demonstrated no association between JIA and *IL32* expression (total *IL32*: mean difference = 0.59, 95% CI −1.91, 3.08; p = 0.63; *IL32*γ: mean difference = −0.00045; 95% CI −0.0051, 0.0042, p = 0.84; *IL32*β: mean difference = 0.060; 95% CI −0.093, 0.21; p = 0.43), although expression of total and β *IL32* was higher in the cases, as might be expected with reduced case methylation. The lack of association between JIA and *IL32* expression was unchanged on removal of the four polyarticular JIA cases from the dataset.

**Figure 5:**
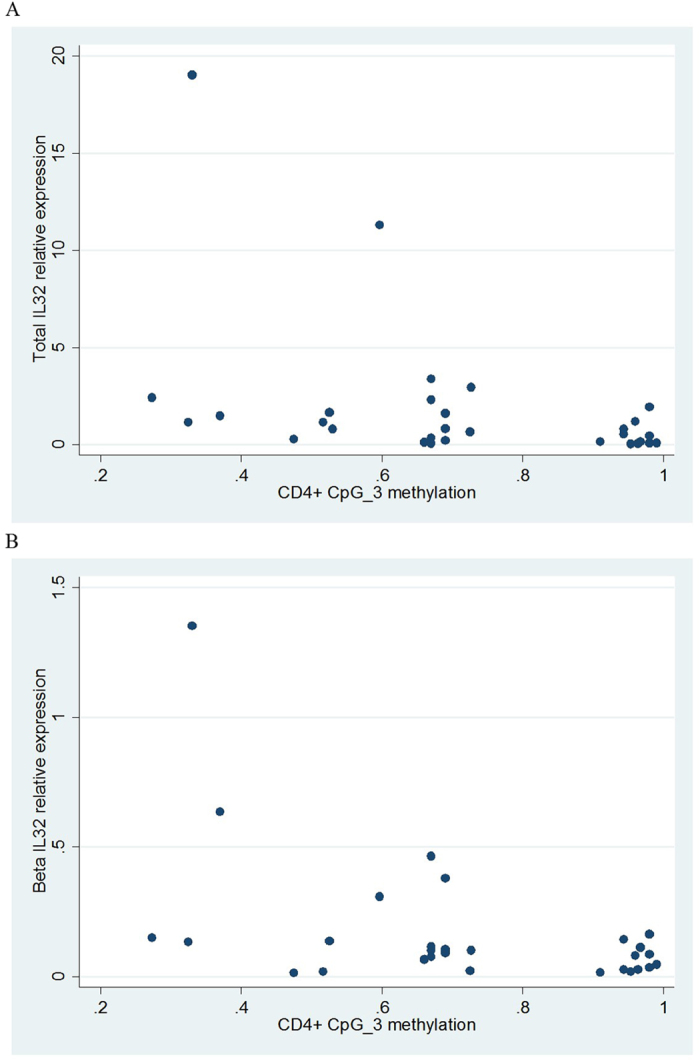
Correlation between *IL32* relative expression and methylation in CD4+ T cells (cases and controls combined). **A**: Total *IL32* and CpG_3, spearman r = −0.73, p = 0.0009. **B**: *IL32*β and CpG_3, spearman r = −0.48, p = 0.049.

We next examined the correlations amongst total, γ and β isoforms in the CD4+ *IL32* combined case-control expression dataset. We observed correlation between total and γ IL32 relative expression (Spearman r = 0.45, p = 0.017) and total and β IL32 relative expression (Spearman r = 0.66, p = 0.0002). There was also a significant correlation between γ and β *IL32* (Spearman r = 0.70, p = < 0.0001). Next, we looked for correlation between CD4+ *IL32* methylation and expression amongst all available samples. There was evidence for a negative correlation between total *IL32* relative expression and CpG_3 (Spearman r = −0.73, p = 0.0009) (Fig. 5A) and *IL32*β relative expression and CpG_3 (Spearman r = −0.48, p = 0.049) (Fig. 5B). In both cases the correlation was in the biologically-predicted direction, that is, expression increased as CpG_3 methylation decreased. We used linear regression to adjust these associations for age, sex and time to blood sample processing and found that the association of total *IL32* with CpG_3 methylation remained significant (β coefficient = −0.071, 95% CI = −0.14, −0.0041, p = 0.039), as did the association between *IL32*β relative expression and CpG_3 methylation (β coefficient = −0.0041, 95%CI = −0.0081, −0.000045, p = 0.048. Methylation levels at no other CpGs were associated with *IL32* expression.

In totality, these analyses do not provide compelling evidence of a direct link between *IL32* expression level and 5’ *IL32* methylation status in CD4+ T cells, or *IL32* gene expression levels and JIA. However, the identification of specific associations in a subset of comparisons warrants further investigation.

### SNPs in the *IL32* gene region are not correlated with *IL32* expression

We did not find any evidence of correlation between *IL32* expression and genotyped SNPs (data not shown).

### Overall relationships

**Figure 6:**
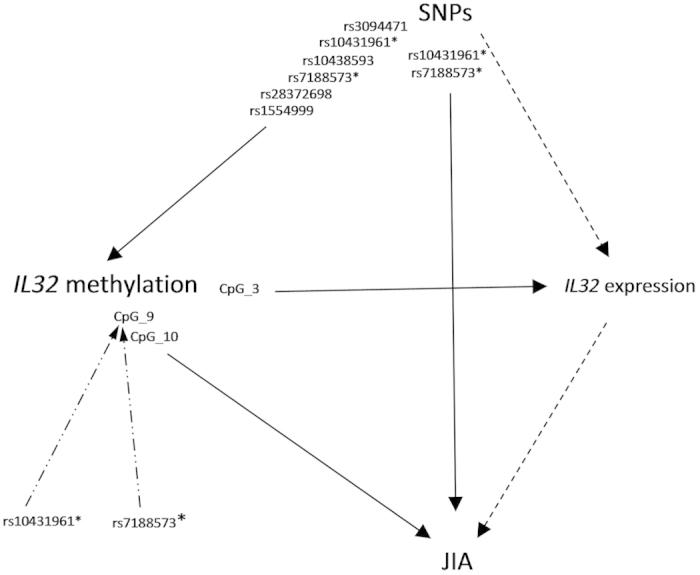
Diagram of the relationships between each of the genomic measures and JIA. Solid lines indicate a correlation/association. Dashed lines indicate no correlation/association. Dot-dash lines indicate interaction such that the minor allele of the SNP potentiates the association between *IL32* methylation and JIA. Asterisks indicate SNPs playing multiple roles in the causal pathway.

The overall relationships of the *IL32* genomic measures amongst themselves, and with JIA, are shown in Fig. 6. Taken together our results show that the association between reduced *IL32* methylation and increased risk of JIA is robust, and that regional genotypes interact with *IL32* 5’ methylation to determine disease risk.

## Discussion

We have confirmed the presence of reduced DNA methylation at CpGs in the 5’ region of *IL32* in circulating T cells in children with oligoarticular course JIA. Our previous data provided evidence, consistent in two case-control samples and across two assay platforms, of reduced *IL32* methylation in JIA CD4+ T cells^20^. Here, we have shown, in a replication case-control sample, a statistically significant association between JIA and *IL32* CpG methylation, with a direction of effect that is consistent with the prior work. When data from the original and replication samples (total 33 case-control pairs) was combined, the presence of association was further confirmed.

To complement these findings, we also measured DNA methylation at *IL32* in CD8+ T cells and found that, in general, this cell type was less methylated than CD4+ T cells. This is consistent with prior evidence that *IL32* is more highly expressed in CD8+ T cells^34^, and suggests a role for *IL32* methylation in *IL32* expression. Given that CD4+ (helper) and CD8+ (cytotoxic) T cells have functionally distinct roles in the immune system, the function of *IL32* may differ between cell types. Indeed, there is evidence that both endogenous and exogenous agents stimulate *IL32* expression in a number of different cell types, suggesting that *IL32* might function in several aspects of immune defense via distinct pathways (for review see^26^). To our knowledge, however, no direct comparison of response of *IL32* to such stimuli between CD4+ and CD8+ T cells has been performed.

There was, however, a significant correlation between CD4+ and CD8+ T cells, and methylation at the key site CpG_9 in CD8+ T cells was also associated with JIA. These data suggest two things. First, the correlation between the two cell types, along with the presence of disease association with both cell types, suggests the establishment of the JIA-related differential methylation patterns in a T cell precursor. To understand how far back in the immune cell lineage this differential methylation pattern extends, further work should examine *IL32* methylation in a wider range of immune cell types. Our data concerning CD4+ and CD8+ T cells suggests that the ‘establishment’ of differential methylation extends at least back prior to maturation to ‘single positive’ T cells in the thymus^35^. This supports a role for differential *IL32* methylation in disease causation, rather than disease consequence, which might more likely be reflected by changes in specific circulating immune cell subsets undertaking distinct functions in relation to disease processes. Correlation of disease-associated methylation changes across multiple immune cell types (T cells, B cells, monocytes) has also been reported in the autoimmune disease systemic lupus erythematosus (SLE)^12^. Such correlation amongst immune cells might provide the potential for DNA methylation to be used as a disease biomarker that is measureable in whole blood, simplifying the assay and thus increasing clinical utility.

By considering both regional SNP genotype and *IL32* gene expression, we have also significantly extended our understanding of the relationships amongst these genomic measures, and how they impact the association between *IL32* methylation and JIA. There is now considerable evidence that genetic sequence variants often impact methylation levels (methylation quantitative trait loci, or mQTLs), particularly early in life^36^,^37^. Data are also emerging implicating genotype-methylation interactions in phenotypic diversity apparent in conditions such as arthritis^9^. Recent data suggest the heritability of methylation profile per se to be around 20%^38^. A number of SNPs genotyped in this study were associated with *IL32* methylation, and associations differed between CpG sites and between T cell types. Of note, rs1554999 was strongly associated with methylation at CpG_10 in CD4+ T cells, and at CpG_9 and CpG_3 in both CD4+ and CD8+ T cells. This SNP is located in the 5’ region of *IL32* (see Fig. 1), 642 bp, 291 bp and 76 bp downstream of CpG_3, CpG_9 and CpG_10 respectively. This SNP has also been strongly associated with *IL32* expression in peripheral blood (see Supplementary Table 3)^39^. This SNP in particular likely represents an *IL32 cis* methylation quantitative trait locus (mQTL). Two SNPs, rs10431961 and rs7188573, which lie 15 kb and 4 kb upstream of the *IL32* transcription start site respectively, were associated both with CD4+ T cell CpG_10 and CpG_9 methylation, and with JIA. Given that JIA is associated with reduced *IL32* methylation, that the minor alleles of the above two SNPs are associated with reduced *IL32* methylation, and that the minor alleles of the two SNPs are associated with an increased risk of JIA in our sample, these SNPs might explain the association between JIA and reduced *IL32* CpG methylation. When these SNPs were included as covariates in the logistic regression model assessing association between JIA and *IL32* methylation, evidence of association between JIA and CD4+ T cell CpG_9 methylation remained. Therefore, genotype at these SNPs does not entirely account for the lower methylation-higher disease risk relationship. Further, we found evidence that these SNPs interact with CpG_9 methylation in determining disease risk, with reduced methylation increasing risk of JIA only in those carrying at least one minor allele. CpG_9 methylation was not associated with JIA in major allele homozygotes. This pattern of association would not be expected if methylation acted only as an intermediary between the SNP and disease risk.

Regulation of gene expression is considered to be one of the major functions of DNA methylation^40^. In general, reduced methylation, particularly in CpG rich gene promoters, is associated with elevated gene expression, although recent evidence suggests that the opposite may be true for some CpG rich sequences, especially those within gene bodies^40^. We therefore assessed the relationship between expression and methylation of *IL32* in CD4+ T cells. We found evidence of an inverse correlation between methylation at CpG_3 and expression of total IL32 and the β *IL32* isoform. However, neither CpG_3 methylation nor *IL32* expression were observed to be associated with JIA.

There are a few potential explanations for the lack of correlation we observed between the JIA-associated differential *IL32* methylation and gene expression. Firstly, other epigenetic mechanisms such as histone modification and chromatin remodelling may be important in regulating gene expression at this locus, obscuring correlation between methylation and expression^41^. Secondly, methylation at CpG sites for which methylation is correlated with expression can lie many kilobases from the relevant gene. It is therefore possible that methylation at the CpGs found to be associated with JIA are more relevant to expression of nearby genes. Genes from both the TNF receptor and matrix metalloproteinase superfamilies lie within 45 kb of CpG_9, and both gene families have been linked to inflammatory diseases^42^,^43^. Thirdly, If *IL32* expression is more dynamic and sensitive to environmental influence than methylation, the impact of sample processing procedures may have served to obscure the methylation – gene expression relationship.

Strengths of our study include careful attention to clinical phenotype to maximise case homogeneity, exclusion of cases treated with methotrexate (a folate inhibitor) and other disease-modifying anti-rheumatic drugs, and consideration of multiple genomic factors and their interactions. A limitation is the relatively small sample size, especially for association of genetic variants with outcomes. Additionally, treatment of cases with corticosteroids was not an exclusion criteria for this study. Although corticosteroids occur naturally in the body, it is possible that corticosteroid treatment (often injected directly to joints) may alter DNA methylation.

In summary, data presented here, in addition to data from our previous work^20^, provides strong evidence that DNA methylation of CpGs in the 5’ region of *IL32* is reduced in oligoarticular JIA. The association was observed in two different immune cell subsets suggestive of a role in determining disease risk. Methylation in this region is impacted by genotype, but the methylation-disease association persists after the effect of genotype is taken into account. In addition, genotype and methylation were seen to interact, with the effect of methylation on JIA risk only evident in those carrying certain alleles. A lack of strong correlation between disease-associated *IL32* methylation and *IL32* expression suggests that other unmeasured factors may need to be considered to elucidate the relationship. Given that *IL32* has an established role in promoting inflammatory arthritis in mouse models and adult rheumatoid arthritis, further work to understand the role of *IL32* in JIA is well justified.

## Materials and methods

### Participant Recruitment and Selection

Cases and controls for the current study were drawn from the ChiLdhood Arthritis Risk factor Identification sTudY (CLARITY). A detailed description of the study has been published previously^44^,^20^. Briefly, cases were recruited from the Royal Children’s Hospital (RCH), Melbourne Australia. Cases were aged 18 years or under at recruitment, and were diagnosed with JIA and classified into clinical subtypes by a paediatric rheumatologist following International League of Associations for Rheumatology (ILAR) criteria^45^. Controls were healthy children aged 16 years or under attending the RCH day surgery unit for a minor surgical procedure. Peripheral blood mononuclear cells (PBMCs) were isolated using a ficoll procedure within 24 hours of blood collection, followed by storage in vapour-phase liquid nitrogen^20^. All study protocols were approved by the RCH Human Research Ethics Committee. Informed consent was obtained from all participants, and the research was carried out in accordance with the approved protocols.

Twenty-one CLARITY JIA cases (17 oligoarticular and four polyarticular) and 21 age and sex matched controls, for whom genome-scale and/or *IL32* locus specific DNA methylation data was previously generated^20^, were included in the current study (‘original’ sample). The sample was augmented by the addition of twelve oligoarticular JIA cases (mean age 4.5 years, SD 3.5 years; 83.33% female) and twelve age and sex matched controls (‘replication’ sample). An additional unpaired case and two unpaired controls, for whom RNA was available, were included for the gene expression analyses (RNA-only sample). All selected cases were naive to methotrexate (MTX) and other biological disease modifying anti-rheumatic drugs at the time of blood collection. The characteristics of all original, replication, and RNA-only cases and controls are shown in Supplementary Table 1.

### T Cell DNA/RNA Isolation

Total viable CD3+ CD4+ T cells, and CD3+ CD8+ T cells (replication samples only) were positively selected for from the PBMC population using flow cytometry (DAPI: Cat ID D9542, Sigma Aldrich, St Louis, MO, USA; CD8-FITC: Cat ID 55536, CD3-APC Cat ID 340440, CD4-PE Cat ID 347327, Beckton Dickinson, San Jose, CA, USA). T cell purities were typically above 98% for CD3+CD4+ T cells and above 95% for CD3+CD8+ following cell sorting. DNA was extracted using the Flexigene DNA extraction kit (Qiagen). Where CD4+ T cell numbers were sufficient, an aliquot of cells was used for RNA extraction using a standard Trizol extraction adapted from Chomczynski, *et al*.^46^, adjusting reagent volume to suit cell numbers. All RNA samples were DNase treated using the Ambion DNA Free kit (Life Technologies, Austin, TX USA).

### Sequenom MassARRAY Epityper IL32 DNA Methylation analysis

DNA was bisulphite converted using the MethylEasy Xceed kit (Human Genetic Signatures, Randwick, NSW Australia) according to the manufacturer’s instructions. Measurement of methylation at *IL32* was performed using two Sequenom MassARRAY Epityper assays as previously described^20^. Figure 1 shows the locations of CpGs in the 5’ region of *IL32*, indicating those measured by the assays.

### Single nucleotide polymorphism (SNP) selection and Sequenom MassARRAY iPlex genotyping

SNPs in the region of *IL32* were selected according to three criteria. First, we used CEU HapMap data^47^ and the tagger function in Haploview^48^ to identify tag-SNPs (defined as a proxy SNP representing a group of one or more SNPs amongst which r^2^ ≥ 0.8) in the region 3kb upstream to 10 kb downstream of the gene. A list of 7 SNPs representing variation at a total of 13 SNPs across the region was generated. Second, we identified any SNPs in the literature that had previously been examined for association with disease outcomes. Third, we used the Genevar eQTL database^49^ to identify SNPs correlated with *IL32* gene expression in either T cells, lymphoblastoid cells or fibroblasts from the Gencord study^50^. We used the Sequenom Assay Design Tool to design a single iPlex multiplex assay that incorporated as many of the identified SNPs as possible. The resulting iPlex assay included a total of 17 SNPs. Rationale for the inclusion of each SNP in the final assay is provided in Supplementary Table 2. SNPs in close proximity to, or within, *IL32* are shown in Fig. 1. Correlations of SNPs with *IL32* expression in the Genevar eQTL database are depicted in Supplementary Figure 1. SNPs also associated with peripheral blood *IL32* expression in the Blood eQTL database^39^ are shown in Supplementary Table 3. SNPs were genotyped using the iPlex chemistry on the Sequenom MassARRAY according to manufacturer’s protocols. Primer sequences are provided in Supplementary Table 4. The Sequenom Typer program was used to call genotypes from raw data in a semi-automated fashion. Outliers on SNP cluster plots were visually inspected and calls rejected if unclear. DNA samples for which genotyping call rate was less than 90% were re-genotyped and subsequently discarded from analysis if call rate remained below 90%.

### Quantitative Real Time PCR Expression Analysis

RNA samples were reverse transcribed to cDNA using the Tetro cDNA synthesis kit (Bioline, London, UK) as per the manufacturer’s instructions. Quantitative real time PCR was then used to quantify expression levels of total *IL32* (all transcript variants), *IL32*γ and *IL32*β. Primers used to measure *IL32*γ and *IL32*β expression were as previously described^23^. Primers used to measure total *IL32* expression were as follows: sense 5’-GATGGATTACGGTGCCGAG-3’; antisense 5’-CACAAAAGCTCTCCCCAGG-3’. *IL32* expression was measured on the Applied Biosystems 7300 Real Time PCR System. Each sample was run in triplicate and a cut-off Cycle Threshold (CT) value of ±1 from the median value of all 3 data points was used to remove outlying replicate values. Replicates that passed this quality control were then used to calculate the mean CT value. The ΔCT analysis method was employed to calculate the relative difference in expression of *IL32* and its isoforms to the endogenous control genes *B2M* (sense 5’-ATCATGGAGGTTTGAAGATGCC**-**3’; antisense 5’- ACATGGAGACAGCACTCAAAGTAGA) and *RPLP0* as per Dheda *et al*^51^.

### Statistical Analysis

We used t-tests (mean comparison tests) and logistic regression to compare methylation β values between cases and controls. We used logistic regression to generate odds ratios (ORs) to gauge the degree of change in risk of JIA with changes in methylation levels. For this purpose we converted methylation β values to % methylation (β x 100) to facilitate OR interpretation. We calculated Pearson correlations amongst methylation data, and between methylation and SNP data. We used linear regression, adjusting for the potential covariates age and sex, to further assess the relationships between methylation and SNPs. We looked for evidence of interaction between SNPs and methylation in relation to JIA risk by use of a product term in logistic regression, adjusting for age and sex. SNP genotype groups were dichotomised for this purpose into major allele homozygotes, and heterozygotes plus minor allele homozygotes (dominant genetic model). For analyses incorporating expression data, we employed non-parametric analyses (Spearman correlation) alongside parametric analyses (linear regression adjusting for the potential covariates age, sex and blood sample time to processing). All analyses were undertaken using Stata v13^52^.

## Additional Information

**How to cite this article**: Meyer, B. *et al*. DNA methylation at *IL32* in juvenile idiopathic arthritis. *Sci. Rep*. **5**, 11063; doi: 10.1038/srep11063 (2015).

## Supplementary Material

Supplementary Information

## Figures and Tables

**Table 1:**
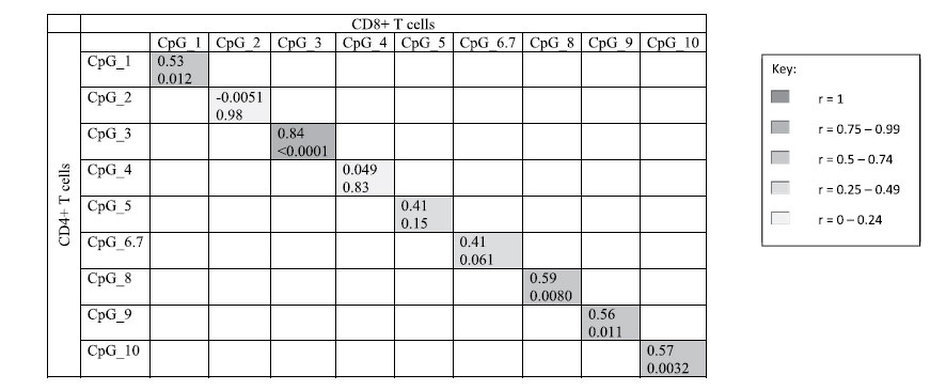
Correlations (Pearson r followed by p-value) between CD4+ and CD8+ T cells for each measured CpG (cases and controls combined).

**Table 2:** Significant associations between genotyped SNPs and *IL32* CpG methylation in CD4+ and CD8+ T cells.

Pearson correlation				Linear regression*			
SNP	CpG	Cell type	Pearson r	p	coefficient	95% CI	p
rs3094471	10	CD4	0.31	0.014	5.57	1.37, 9.77	0.010
	3	CD4	0.30	0.019	9.18	1.15, 17.21	0.026
rs10431961	10	CD4	−0.29	0.020	−5.29	−9.49, −1.09	0.015
	9	CD4	−0.40	0.0018	−11.27	−17.62, −4.93	0.001
	5	CD4	−0.27	0.048	−6.32	−11.39, −1.26	0.015
	10	CD8	−0.42	0.041	−4.39	−8.15, −0.62	0.025
rs10438593	10	CD4	−0.32	0.010	−7.75	−13.44, −2.06	0.008
	9	CD4	−0.43	0.0008	−16.61	−24.73, −8.48	<0.001
rs7188573	10	CD4	−0.32	0.012	−6.17	−10.64, −1.71	0.008
	9	CD4	−0.44	0.0005	−12.77	−19.23, −6.32	<0.001
	6.7	CD4	−0.28	0.033	−4.17	−7.22, −1.13	0.008
	5	CD4	−0.33	0.016	−7.65	−12.74, −2.55	0.004
	10	CD8	−0.59	0.0025	−8.02	−11.83, −4.21	<0.001
	9	CD8	−0.40	0.059	−10.20	−18.87, −1.53	0.024
rs28372698	10	CD4	−0.27	0.030	−4.21	−8.05, −0.36	0.032
	3	CD4	−0.76	<0.0001	−21.6	−26.56, −16.64	<0.001
	3	CD8	−0.63	0.0011	−17.21	−26.20, −8.21	0.001
rs1554999	10	CD4	0.54	<0.0001	8.71	5.44, 11.99	<0.001
	9	CD4	0.60	<0.0001	14.09	9.20, 18.99	<0.001
	3	CD4	0.54	<0.0001	16.10	9.74, 22.46	<0.001
	9	CD8	0.43	0.040	8.52	1.71, 15.34	0.017
	3	CD8	0.66	0.0004	21.78	13.28, 30.29	<0.001
rs2239302	6.7	CD8	−0.45	0.027	−14.93	−29.04, −0.83	0.039
rs3789033	4	CD8	−0.44	0.033	−7.56	−13.96, −1.15	0.023
	2	CD8	−0.67	0.0008	−4.20	−7.17, −1.23	0.008
rs2239316	4	CD8	−0.44	0.034	−6.84	−12.65, −1.03	0.023
	2	CD8	−0.63	0.0020	−3.43	−6.22, −0.64	0.019

^*^For linear regression, CpG methylation was converted to % methylation (β*100) for easier interpretation of the coefficient. The coefficient was adjusted for age and sex. The coefficient represents the change in % methylation for every additional minor allele of the SNP.
