# Supplementary material for: DNA methylation at IL32 in juvenile idiopathic arthritis
Source: Sci Rep. 2015 Jun 9;5:11063. doi: 10.1038/srep11063 (PMC4603785; doi:10.1038/srep11063)
Supplement: Supplementary Information [file srep11063-s1.pdf]

**<u>Supplementary File</u>**

# DNA methylation at *IL32* in juvenile idiopathic arthritis

Braydon Meyer, Raul A Chavez, Jane E Munro, Rachel C Chiaroni-Clarke, Jonathan D Akikusa, Roger C Allen, Jeffrey M Craig, Anne-Louise Ponsonby, Richard Saffery, Justine A Ellis

**Supplementary Table 1.** Characteristics of cases and controls included in the current study.

| Case/Control Pair Number | Case Participant ID | Control Participant ID | Pair Age | Pair Sex | Case JIA Subtype |
|---|---|---|---|---|---|
| Previously analysed 'original' samples | | | | | |
| 1 | J01 | C01 | 2 | F | Oligoarticular |
| 2 | J02 | C02 | 5 | F | Polyarticular |
| 3 | J03 | C03 | 7 | F | Oligoarticular |
| 4 | J04 | C04 | 9 | F | Oligoarticular |
| 5 | J05 | C05 | 11 | F | Oligoarticular |
| 6 | J06 | C06 | 12 | M | Oligoarticular |
| 7 | J07 | C07 | 12 | M | Oligoarticular |
| 8 | J08 | C08 | 11 | M | Polyarticular |
| 9 | J09 | C09 | 12 | F | Oligoarticular |
| 10 | J10 | C10 | 14 | M | Oligoarticular |
| 11 | J11 | C11 | 1 | M | Oligoarticular |
| 12 | J12 | C12 | 3 | F | Oligoarticular |
| 13 | J13 | C13 | 6 | F | Oligoarticular |
| 14 | J14 | C14 | 8 | F | Oligoarticular |
| 15 | J15 | C15 | 12 | F | Oligoarticular |
| 16 | J16 | C16 | 14 | F | Oligoarticular |
| 17 | J17 | C17 | 6 | F | Oligoarticular |
| 18 | J18 | C18 | 12 | M | Oligoarticular |
| 19 | J19 | C19 | 11 | F | Polyarticular |
| 20 | J20 | C20 | 2 | F | Oligoarticular |
| 21 | J21 | C21 | 3 | F | Polyarticular |
| New 'replication' samples | | | | | |
| 22 | J22 | C22 | 12 | F | Oligoarticular |
| 23 | J23 | C23 | 3 | F | Oligoarticular |
| 24 | J24 | C24 | 6 | M | Oligoarticular |
| 25 | J25 | C25 | 2 | F | Oligoarticular |
| 26 | J26 | C26 | 1 | F | Oligoarticular |
| 27 | J27 | C27 | 10 | F | Oligoarticular |
| 28 | J28 | C28 | 1 | F | Oligoarticular |
| 29 | J29 | C29 | 2 | M | Oligoarticular |
| 30 | J30 | C30 | 4 | F | Oligoarticular |
| 31 | J31 | C31 | 3 | F | Oligoarticular |
| 32 | J32 | C32 | 3 | F | Oligoarticular |
| 33 | J33 | C33 | 7 | F | Oligoarticular |
| Unpaired 'RNA only' samples | | | | | |
| - | J34 | | 9 | M | Oligoarticular |
| - | - | C35 | 12 | F | - |
| - | - | C36 | 11 | M | - |

Previously analysed ‘original’ samples = samples included in previous genome-scale DNA methylation study [1]

New ‘replication’ samples = samples not used previously and new to this study

Unpaired ‘RNA only’ samples = samples not within a case-control pair in whom RNA was available for gene expression analysis.

Oligoarticular = oligoarticular disease course.

Polyarticular = polyarticular RF negative disease course.

**Supplementary Table 2**: Rationale for the selection of each genotyped SNP

| SNP | Chr 16 coordinate* | Reason for inclusion |
|---|---|---|
| rs3094471 | 2693181 | Genevar |
| rs10431961 | 3040096 | Genevar |
| rs10438593 | 3050715 | Literature [2] |
| rs7188573 | 3051204 | Literature [2] |
| rs28372698 | 3055112 | HapMap/Literature [3] |
| rs1554999 | 3055629 | HapMap/Literature [2] |
| rs1555001 | 3057355 | Literature [2] |
| rs2239302 | 3057878 | HapMap |
| rs12934561 | 3058866 | Literature [4] |
| rs4786376 | 3065152 | HapMap |
| rs40222 | 3429645 | Genevar |
| rs6501178 | 3506363 | Genevar |
| rs11076781 | 3603232 | Genevar |
| rs740862 | 3629679 | Genevar |
| rs3789033 | 3779536 | Genevar |
| rs2239316 | 3853996 | Genevar |
| rs2238439 | 3971349 | Genevar |

*hg18 human genome build

Genevar = Correlated with *IL32* expression in Genevar database

Literature = previously examined in the literature

HapMap = selected as a tag-SNP using HapMap CEU data

**Supplementary Figure 1:** Genevar eQTL database prior evidence of association of genotyped SNPs with IL32 expression. Where association was seen for a SNP with IL32 expression using more than one expression array probe or tissue type, the most significant is shown. A. rs3094471, GenCord-T cells. B. rs10431961, GenCord fibroblast cells. C. rs40222, GenCord fibroblast cells. D. rs6501178, GenCord T cells. E. rs11076781, GenCord T cells. F. rs740862, GenCord T cells. G. rs3789033, GenCord fibroblast cells. H. rs2239316, GenCord lymphoblast cells. I. rs2238439, GenCord fibroblast cells.
Data drawn from [5].

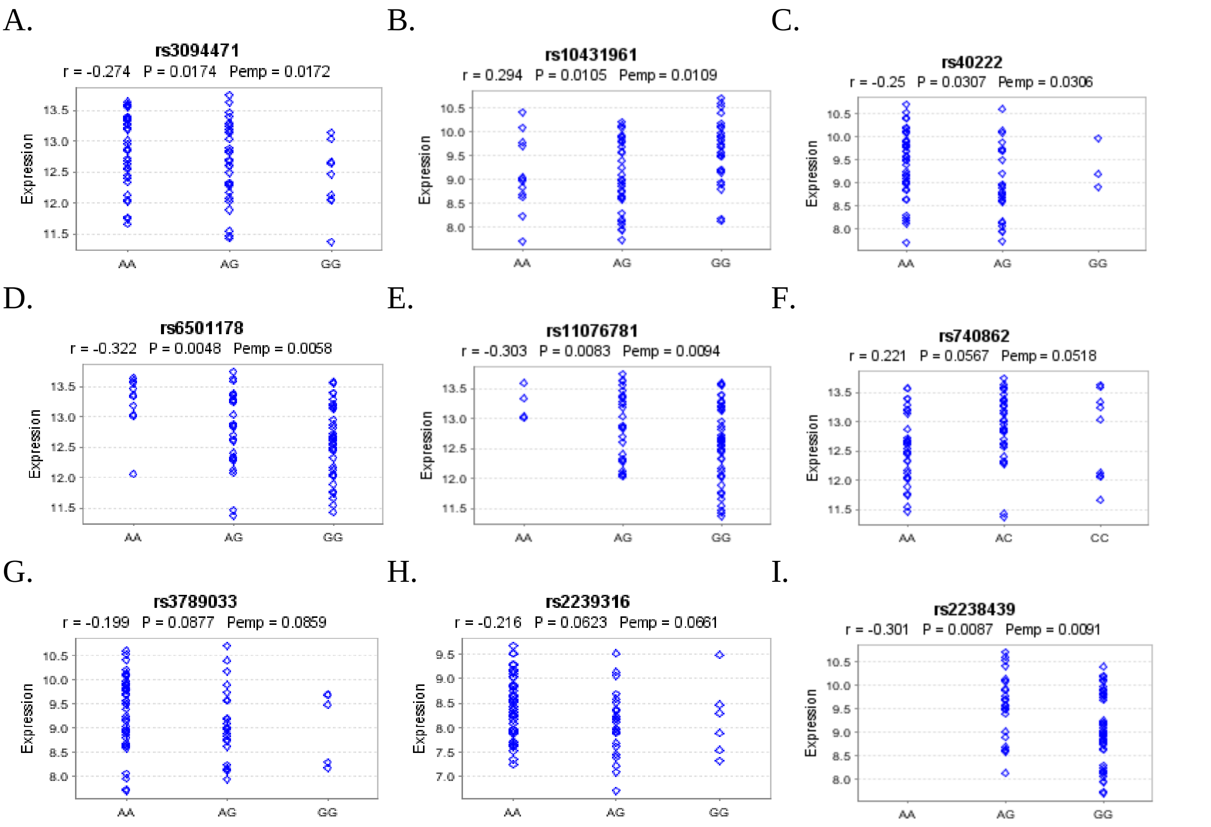

**Supplementary Table 3:** Evidence of association of genotyped SNPs with IL32 expression in peripheral blood taken directly from the Blood eQTL database (http://genenetwork.nl/bloodeqtlbrowser/) [6]

| SNP | SNP Chr. | SNP Chr. Position* | Probe[§] | Probe Chr. | Probe Chr. Position* | SNP Alleles | Minor Allele | Z-score | Gene name | P-value |
|---|---|---|---|---|---|---|---|---|---|---|
| rs10431961 | 16 | 3040096 | 5910112 | 16 | 3059592 | C/T | T | 13.27 | IL32 | 3.36E-40 |
| rs10431961 | 16 | 3040096 | 3440754 | 16 | 3058208 | C/T | T | 12.92 | IL32 | 3.34E-38 |
| rs10438593 | 16 | 3050715 | 5910112 | 16 | 3059592 | C/T | T | 10.46 | IL32 | 1.27E-25 |
| rs10438593 | 16 | 3050715 | 3440754 | 16 | 3058208 | C/T | T | 9.71 | IL32 | 2.76E-22 |
| rs7188573 | 16 | 3051204 | 5910112 | 16 | 3059592 | T/C | C | 15.77 | IL32 | 4.80E-56 |
| rs7188573 | 16 | 3051204 | 3440754 | 16 | 3058208 | T/C | C | 15.25 | IL32 | 1.69E-52 |
| rs1554999 | 16 | 3055629 | 5910112 | 16 | 3059592 | C/A | A | -26.35 | IL32 | 4.92E-153 |
| rs1554999 | 16 | 3055629 | 3440754 | 16 | 3058208 | C/A | A | -26.09 | IL32 | 4.45E-150 |
| rs2239302 | 16 | 3057878 | 3440754 | 16 | 3058208 | G/A | A | 18.82 | IL32 | 5.54E-79 |
| rs2239302 | 16 | 3057878 | 5910112 | 16 | 3059592 | G/A | A | 18.08 | IL32 | 4.89E-73 |

* hg18 genome build

[§] Illumina whole genome expression BeadChips (HT12v3, HT12v4 or H8v2)

**Supplementary Table 4:** Sequenom iPlex SNP primer sequences for the SNPs genotyped in this study

| SNP ID | Forward Primer Sequence | Reverse Primer Sequence | Extended Primer Sequence |
|---|---|---|---|
| rs10431961 | ACGTTGGATGTGCTCCCTGCCTGACGTGCT | ACGTTGGATGATGTCAGGGTTCGCTTCTTC | GGTCAGGCGGCGTCG |
| rs4786376 | ACGTTGGATGGTGACAAGGGTGCAGGAAG | ACGTTGGATGTCTCTCACCTTCTATCCTGC | CTATCCTGCCTTGCAT |
| rs1554999 | ACGTTGGATGCCCACTTTCCCCATAAAACC | ACGTTGGATGAGACAGTCACCTTCTGCACG | GTCTTCCTGGCACAAA |
| rs2239302 | ACGTTGGATGAAACAACAGGGGTGCCAGAC | ACGTTGGATGACATCAGTCAGTCCTCAAGC | aaCGGGCCCCTGGCTGG |
| rs1555001 | ACGTTGGATGATTATCCTGGAGGAAAGGTT | ACGTTGGATGTCTTCATGTCATCAGAGAGG | AGGTGACACATGGAGAC |
| rs7188573 | ACGTTGGATGAGAAAGAAGAGTGGGAGGAC | ACGTTGGATGAGTCTCAATTCCACAGTGGC | CCCATAACCAACTTTTCG |
| rs28372698 | ACGTTGGATGTATCTTGTCCCACAGGTAG | ACGTTGGATGAGGAACTGCCGGACCTAAGA | CCCACAGGTAGATCTACA |
| rs11076781 | ACGTTGGATGTCACTTATCGGCTTTCTGTC | ACGTTGGATGAAAGCCCAGCAGAAAAAGCC | AGCAGAAAAAGCCAAGTAT |
| rs2239316 | ACGTTGGATGGTTGTTTACCACCACCACAG | ACGTTGGATGGAGAATCCTCTCCCTGTAAG | accaTGTAAGCAGGGTGGA |
| rs3094471 | ACGTTGGATGCCTCAACCTTAAGAACGTGC | ACGTTGGATGCCTGCCTCGTTTGCTTTCAG | gcttCCCACAGGTGACCCAT |
| rs10438593 | ACGTTGGATGACTTATTCATCCCACTCAGG | ACGTTGGATGGGTCCATCAATGCTCAGAAG | CCCACTCAGGACAATAAAAA |
| rs12934561 | ACGTTGGATGTCTGGGAAAAGTCCCTCTTG | ACGTTGGATGACGGAGGCCCACAGGTGTT | gacaACAGGTGTTGGTTTCCC |
| rs3789033 | ACGTTGGATGACTGACTTGTCTCTACCTCG | ACGTTGGATGCCTACGTTGGCTTAAGGTTG | GTTGGGTTTTGAAGACAATAT |
| rs2238439 | ACGTTGGATGAAGGAGCAGGGAATAATAGC | ACGTTGGATGTAATGTGGGTAGCTGTAGCG | AAGTAAAAAGGAATGGAGACA |
| rs740862 | ACGTTGGATGTTCCCTCTTAGTCAACTCCC | ACGTTGGATGTCCCACCAGGGGACAGAAAC | aTTATGCTAAACTTCCCTTGTT |
| rs40222 | ACGTTGGATGTGATCTGAACCTATCCTAAC | ACGTTGGATGTGTTGTGGGAGGCAGTTAAG | cGGAGTCAGTTTGACTGTTTCA |
| rs6501178 | ACGTTGGATGCAAGAATTTGCTATGAGTTG | ACGTTGGATGGCCCAGCAGGTATTACTGTT | ACTGTTTAATGACAAAAGAATTA |

**Supplementary Table 5**: *IL32* CpG methylation beta values in CD4+ and CD8+ T cells from ‘replication’ cases and controls

| | | Sequenom assay unit | | | | | | | | | | | | | | | | | |
|---|---|---|---|---|---|---|---|---|---|---|---|---|---|---|---|---|---|---|---|
| | | CpG_1 | | CpG_2 | | CpG_3 | | CpG_4 | | CpG_5 | | CpG_6.7 | | CpG_8 | | CpG_9 | | CpG_10 | |
| Pair # | Sample type | CD4 | CD8 | CD4 | CD8 | CD4 | CD8 | CD4 | CD8 | CD4 | CD8 | CD4 | CD8 | CD4 | CD8 | CD4 | CD8 | CD4 | CD8 |
| 22 | case | 0.84 | 0.76 | 0.83 | 0.84 | 0.27 | 0.34 | 0.78 | 0.76 | 0.41 | 0.37 | 0.74 | 0.74 | 0.77 | 0.69 | 0.15 | 0.07 | 0.067 | 0.030 |
| | *control* | *0.94* | *0.69* | *0.97* | *0.82* | *0.69* | *0.68* | *0.57* | *0.62* | - | *0.41* | *1.00* | *0.63* | *0.88* | *0.52* | - | *0.18* | *0.050* | *0.060* |
| 23 | case | 0.97 | 0.72 | - | 0.82 | 0.97 | 0.98 | 0.52 | 0.72 | - | 0.26 | - | 0.69 | - | 0.67 | - | 0.31 | 0.093 | 0.020 |
| | *control* | - | *0.77* | - | *0.82* | - | *0.94* | - | *0.75* | - | *0.35* | *0.99* | *0.74* | - | *0.70* | *0.78* | *0.21* | *0.33* | *0.070* |
| 24 | case | 0.76 | 0.53 | 0.84 | - | 0.94 | 0.60 | 0.76 | 0.41 | 0.44 | - | 0.69 | 0.41 | 0.64 | 0.35 | 0.11 | 0.070 | 0.030 | 0.010 |
| | *control* | *0.96* | *0.70* | *0.90* | - | *0.96* | *0.89* | *0.87* | *0.68* | *0.74* | *0.47* | *0.92* | *0.72* | *0.86* | *0.72* | *0.61* | *0.43* | *0.38* | *0.24* |
| 25 | case | 0.95 | 0.90 | 0.91 | 0.92 | 0.53 | 0.51 | 0.85 | 0.75 | 0.72 | 0.70 | 0.91 | 0.86 | 0.87 | 0.85 | 0.28 | 0.15 | 0.060 | 0.030 |
| | *control* | *0.89* | *0.79* | *0.88* | *0.89* | *0.96* | *0.97* | *0.81* | *0.79* | *0.53* | *0.43* | *0.81* | *0.75* | *0.75* | *0.74* | *0.25* | *0.21* | *0.12* | *0.080* |
| 26 | case | 0.94 | 0.97 | 0.94 | 0.92 | 0.52 | 0.51 | 0.80 | 0.68 | 0.72 | - | 0.90 | 0.88 | 0.86 | 0.70 | 0.33 | 0.13 | 0.087 | 0.070 |
| | *control* | *1.00* | *0.84* | *0.92* | *0.87* | *0.69* | *0.66* | *0.83* | *0.73* | *0.94* | *0.50* | *0.95* | *0.78* | *0.91* | *0.76* | *0.55* | *0.36* | *0.22* | *0.15* |
| 27 | case | 0.85 | 0.75 | 0.88 | 0.83 | 0.97 | 0.93 | 0.79 | 0.72 | 0.46 | 0.27 | 0.74 | 0.64 | 0.74 | 0.58 | 0.18 | 0.070 | 0.050 | 0.010 |
| | *control* | *0.98* | *0.99* | *0.91* | - | *0.60* | *0.51* | *0.84* | *0.53* | *0.51* | - | *0.83* | *0.93* | *0.74* | - | *0.40* | *0.12* | *0.20* | *0.13* |
| 28 | case | 0.93 | 0.87 | 0.91 | 0.89 | 0.69 | 0.71 | 0.84 | 0.77 | 0.72 | 0.52 | 0.89 | 0.79 | 0.86 | 0.72 | 0.46 | 0.19 | 0.25 | 0.080 |
| | *control* | *0.97* | *0.90* | *0.89* | *0.88* | *0.95* | *0.66* | *0.80* | *0.87* | *0.88* | *0.53* | *0.93* | *0.83* | *0.89* | *0.83* | *0.65* | *0.18* | *0.29* | *0.060* |
| 29 | case | 0.92 | 0.90 | 0.93 | 0.92 | 0.73 | 0.99 | 0.82 | 0.80 | 0.75 | - | 0.91 | 0.92 | 0.86 | 0.91 | 0.51 | - | 0.16 | 0.19 |
| | *control* | *0.92* | *0.71* | *0.89* | *0.88* | *0.98* | *0.93* | *0.65* | *0.69* | - | *0.34* | *0.93* | *0.81* | *0.84* | *0.75* | *0.49* | *0.25* | *0.097* | *0.090* |
| 30 | case | 0.89 | 0.86 | 0.91 | 0.89 | 0.67 | 0.63 | 0.81 | 0.81 | 0.55 | 0.54 | 0.83 | 0.86 | 0.81 | 0.82 | 0.24 | 0.11 | 0.10 | 0.030 |
| | *control* | *0.85* | *0.88* | *0.81* | *0.88* | *0.94* | *0.96* | *0.70* | *0.82* | *0.43* | *0.66* | *0.77* | *0.86* | *0.73* | *0.81* | *0.38* | *0.47* | *0.15* | *0.26* |
| 31 | case | - | 0.89 | 0.94 | 0.84 | 0.96 | 0.89 | 0.55 | 0.76 | - | 0.43 | - | 0.73 | - | 0.81 | - | 0.090 | 0.005 | 0.050 |
| | *control* | *0.99* | *0.83* | *0.94* | *0.75* | *0.69* | *0.60* | *0.77* | *0.67* | - | *0.44* | *0.89* | *0.67* | *0.82* | - | *0.40* | *0.25* | *0.16* | *0.090* |
| 32 | case | 0.96 | 0.78 | 0.90 | 0.86 | 0.97 | 0.94 | 0.80 | 0.74 | 0.64 | 0.44 | 0.87 | 0.67 | 0.82 | 0.63 | 0.30 | 0.16 | 0.14 | 0.050 |
| | *control* | *0.81* | *0.70* | *0.87* | *0.80* | *0.52* | *0.49* | *0.78* | *0.68* | *0.47* | *0.22* | *0.72* | *0.59* | *0.66* | *0.53* | *0.13* | *0.070* | *0.027* | *0.030* |
| 33 | case | 0.80 | 0.75 | 0.84 | 0.84 | 0.94 | 0.98 | 0.78 | 0.65 | 0.52 | 0.42 | 0.77 | 0.66 | 0.73 | 0.68 | 0.38 | 0.33 | 0.19 | 0.14 |
| | *control* | *0.90* | *0.83* | *0.90* | *0.85* | *0.97* | *0.91* | *0.76* | *0.76* | *0.59* | *0.53* | *0.84* | *0.78* | *0.86* | *0.70* | *0.41* | *0.34* | *0.27* | *0.14* |
| | | | | | | | | | | | | | | | | | | | |
| | case mean (SD) | 0.89 (0.021) | 0.81 (0.034) | 0.89 (0.012) | 0.87 (0.011) | 0.76 (0.067) | 0.75 (0.066) | 0.76 (0.032) | 0.71 (0.031) | 0.59 (0.042) | 0.44 (0.046) | 0.82 (0.026) | 0.74 (0.040) | 0.80 (0.024) | 0.70 (0.042) | 0.29 (0.041) | 0.15 (0.027) | 0.10 (0.021) | 0.059 (0.015) |
| | control mean (SD) | 0.93 (0.018) | 0.80 (0.027) | 0.90 (0.012) | 0.84 (0.014) | 0.81 (0.053) | 0.77 (0.053) | 0.76 (0.027) | 0.71 (0.027) | 0.64 (0.068) | 0.44 (0.035) | 0.88 (0.025) | 0.76 (0.028) | 0.81 (0.024) | 0.71 (0.033) | 0.46 (0.055) | 0.26 (0.035) | 0.19 (0.032) | 0.12 (0.021) |
| | t-test p | 0.21 | 0.97 | 0.76 | 0.17 | 0.56 | 0.86 | 0.94 | 0.99 | 0.56 | 0.93 | 0.14 | 0.69 | 0.67 | 0.88 | 0.028 | 0.034 | 0.032 | 0.037 |

**Supplementary Table 6**: Correlation matrix (Pearson r followed by p-value) for all measured CpGs in CD4+ T cells ('original' and replication' cases and controls combined)

| | CpG_1 | CpG_2 | CpG_3 | CpG_4 | CpG_5 | CpG_6.7 | CpG_8 | CpG_9 | CpG_10 |
|---|---|---|---|---|---|---|---|---|---|
| CpG_1 | 1.00 | | | | | | | | |
| CpG_2 | 0.71<br><0.0001 | 1.00 | | | | | | | |
| CpG_3 | 0.21<br>0.10 | 0.089<br>0.50 | 1.00 | | | | | | |
| CpG_4 | 0.22<br>0.10 | 0.10<br>0.45 | -0.11<br>0.42 | 1.00 | | | | | |
| CpG_5 | 0.80<br><0.0001 | 0.56<br><0.0001 | 0.15<br>0.27 | 0.63<br><0.0001 | 1.00 | | | | |
| CpG_6.7 | 0.84<br><0.0001 | 0.67<br><0.0001 | 0.21<br>0.10 | 0.21<br>0.12 | 0.86<br><0.0001 | 1.00 | | | |
| CpG_8 | 0.80<br><0.0001 | 0.55<br><0.0001 | 0.23<br>0.078 | 0.39<br>0.0030 | 0.84<br><0.0001 | 0.88<br><0.0001 | 1.00 | | |
| CpG_9 | 0.59<br><0.0001 | 0.40<br>0.0025 | 0.36<br>0.062 | 0.13<br>0.33 | 0.72<br><0.0001 | 0.69<br><0.0001 | 0.68<br><0.0001 | 1.00 | |
| CpG_10 | 0.37<br>0.0036 | 0.17<br>0.20 | 0.38<br>0.0024 | 0.31<br>0.016 | 0.57<br><0.0001 | 0.44<br>0.0004 | 0.53<br><0.0001 | 0.86<br><0.0001 | 1.00 |

Key:
- r = 1
- r = 0.75 – 0.99
- r = 0.5 – 0.74
- r = 0.25 – 0.49
- r = 0 – 0.24

**Supplementary Table 7**: Correlation matrix (Pearson r followed by p-value) for all measured CpGs in CD8+ T cells ('replication' cases and controls combined)

| | CpG_1 | CpG_2 | CpG_3 | CpG_4 | CpG_5 | CpG_6.7 | CpG_8 | CpG_9 | CpG_10 |
|---|---|---|---|---|---|---|---|---|---|
| CpG_1 | 1.00 | | | | | | | | |
| CpG_2 | 0.58<br>0.0062 | 1.00 | | | | | | | |
| CpG_3 | -0.14<br>0.52 | 0.043<br>0.85 | 1.00 | | | | | | |
| CpG_4 | 0.48<br>0.018 | 0.50<br>0.022 | 0.28<br>0.19 | 1.00 | | | | | |
| CpG_5 | 0.76<br>0.0001 | 0.61<br>0.0052 | -0.11<br>0.64 | 0.49<br>0.030 | 1.00 | | | | |
| CpG_6.7 | 0.86<br><0.0001 | 0.82<br><0.0001 | 0.0003<br>1.00 | 0.55<br>0.0055 | 0.76<br>0.0001 | 1.00 | | | |
| CpG_8 | 0.81<br><0.0001 | 0.73<br>0.0003 | 0.21<br>0.35 | 0.83<br><0.0001 | 0.72<br>0.0005 | 0.92<br><0.0001 | 1.00 | | |
| CpG_9 | 0.017<br>0.94 | 0.010<br>0.97 | 0.54<br>0.0079 | 0.22<br>0.31 | 0.35<br>0.13 | 0.21<br>0.34 | 0.34<br>0.13 | 1.00 | |
| CpG_10 | 0.25<br>0.23 | 0.23<br>0.32 | 0.38<br>0.069 | 0.13<br>0.56 | 0.44<br>0.054 | 0.41<br>0.049 | 0.41<br>0.060 | 0.84<br><0.0001 | 1.00 |

Key:
- r = 1
- r = 0.75 – 0.99
- r = 0.5 – 0.74
- r = 0.25 – 0.49
- r = 0 – 0.24

**Supplementary Table 8**: IL32 expression data in cases and controls for whom RNA was available.

| Pair Number | Sample Type | Participant ID | Total IL32 ΔCT | | IL32 Gamma ΔCT | | IL32 Beta ΔCT | |
|---|---|---|---|---|---|---|---|---|
| | | | dCt | Relative expression | dCt | Relative expression | dCt | Relative expression |
| 12 | Case | J00329 | 1.216 | 2.324 | -8.365 | 0.003 | -3.302 | 0.101 |
| | Control | C00106 | -0.603 | 0.658 | -10.26 | 0.001 | -5.542 | 0.021 |
| 15 | Case | J00319 | 0.21 | 1.157 | -9.335 | 0.002 | -2.895 | 0.134 |
| | Control | C00512 | 1.756 | 3.378 | -6.595 | 0.01 | -1.103 | 0.466 |
| 16 | Case | J00344 | 0.575 | 1.49 | -4.53 | 0.043 | -0.653 | 0.636 |
| | Control | C00494 | -2.779 | 0.146 | -9.172 | 0.002 | -5.977 | 0.016 |
| 17 | Case | J00136 | -1.162 | 0.447 | -8.226 | 0.003 | -2.612 | 0.164 |
| | Control | C00290 | 0.734 | 1.663 | - | - | -2.873 | 0.137 |
| 18 | Case | J00182 | -1.601 | 0.33 | -8.236 | 0.003 | -3.112 | 0.116 |
| | Control | C00123 | -0.286 | 0.82 | -5.161 | 0.028 | -1.398 | 0.379 |
| 19 | Case | J00325 | 4.251 | 19.034 | -5.154 | 0.028 | 0.435 | 1.352 |
| | Control | C00127 | -1.83 | 0.281 | -9.538 | 0.001 | -6.145 | 0.014 |
| 20 | Case | J00342 | -3.463 | 0.091 | -8.669 | 0.002 | -4.86 | 0.034 |
| | Control | C00184 | -3.428 | 0.093 | -7.526 | 0.005 | -4.375 | 0.048 |
| 21 | Case | J00091 | -0.853 | 0.554 | -9.908 | 0.001 | -5.809 | 0.018 |
| | Control | C00413 | -2.886 | 0.135 | -8.115 | 0.004 | -3.888 | 0.068 |
| 22 | Case | J00297 | 1.273 | 2.416 | -7.761 | 0.005 | -2.733 | 0.15 |
| | Control | - | - | - | - | - | | |
| 24 | Case | J00354 | -0.31 | 0.806 | -7.985 | 0.004 | -2.8 | 0.144 |
| | Control | C00364 | -3.997 | 0.063 | - | - | -5.234 | 0.027 |
| 25 | Case | J00395 | -0.322 | 0.8 | -8.373 | 0.003 | - | - |
| | Control | C00540 | 0.252 | 1.191 | -7.552 | 0.005 | -3.618 | 0.081 |
| 26 | Case | J00343 | 0.202 | 1.15 | -9.254 | 0.002 | -5.699 | 0.019 |
| | Control | C00035 | 0.687 | 1.61 | -8.443 | 0.003 | -3.25 | 0.105 |

| | | | | | | | | |
|---|---|---|---|---|---|---|---|---|
| 27 | Case | J00181 | -2.672 | 0.157 | - | - | -3.153 | 0.112 |
| | Control | C00306 | 3.499 | 11.304 | -8.181 | 0.003 | -1.694 | 0.309 |
| 28 | Case | J00242 | -2.316 | 0.201 | -8.18 | 0.003 | -3.464 | 0.091 |
| | Control | C00409 | -4.831 | 0.035 | -10.933 | 0.001 | -5.673 | 0.02 |
| 29 | Case | J00401 | 1.558 | 2.944 | -8.698 | 0.002 | -3.291 | 0.102 |
| | Control | C00433 | 0.956 | 1.94 | -7.713 | 0.005 | -3.54 | 0.086 |
| 30 | Case | J00116 | -4.189 | 0.055 | - | - | -3.697 | 0.077 |
| | Control | C00242 | -0.918 | 0.529 | - | - | -5.251 | 0.026 |
| Unpaired | Case | J00346 | 0.888 | 1.851 | -8.267 | 0.003 | -3.598 | 0.083 |
| | Control | C00475 | 3.278 | 9.701 | -6.186 | 0.014 | -0.354 | 0.782 |
| | Control | C00516 | 1.247 | 2.373 | -7.139 | 0.007 | -3.421 | 0.093 |
| | Case Mean | | | 2.106 | | 0.007 | | 0.158 |
| | Case SD | | | 4.452 | | 0.012 | | 0.210 |
| | Case range | | | 0.055-19.03 | | 0.001-0.043 | | 0.018-1.35 |
| | Control Mean | | | 2.113 | | 0.006 | | 0.208 |
| | Control SD | | | 3.308 | | 0.007 | | 0.336 |
| | Control range | | | 0.035–11.30 | | 0.0005-0.028 | | 0.014-0.78 |
| | t-test p | | | 0.99 | | 0.82 | | 0.60 |

**Supplementary references**


1. Ellis JA, Munro JE, Chavez RA, Gordon L, Joo JE, Akikusa JD *et al.* Genome-scale case-control analysis of CD4+ T-cell DNA methylation in juvenile idiopathic arthritis reveals potential targets involved in disease. *Clinical epigenetics* 2012; **4**(1)**:** 20.

2. Lehtinen AB, Cox AJ, Ziegler JT, Voruganti VS, Xu J, Freedman BI *et al.* Genetic mapping of vascular calcified plaque loci on chromosome 16p in European Americans from the diabetes heart study. *Annals of human genetics* 2011; **75**(2)**:** 222-35.

3. Plantinga TS, Costantini I, Heinhuis B, Huijbers A, Semango G, Kusters B *et al.* A promoter polymorphism in human interleukin-32 modulates its expression and influences the risk and the outcome of epithelial cell-derived thyroid carcinoma. *Carcinogenesis* 2013; **34**(7)**:** 1529-35.

4. Arcaroli JJ, Liu N, Yi N, Abraham E. Association between IL-32 genotypes and outcome in infection-associated acute lung injury. *Critical care* 2011; **15**(3)**:** R138.

5. Dimas AS, Deutsch S, Stranger BE, Montgomery SB, Borel C, Attar-Cohen H *et al.* Common regulatory variation impacts gene expression in a cell type-dependent manner. *Science* 2009; **325**(5945)**:** 1246-50.

6. Westra HJ, Peters MJ, Esko T, Yaghootkar H, Schurmann C, Kettunen J *et al.* Systematic identification of trans eQTLs as putative drivers of known disease associations. *Nature genetics* 2013; **45**(10)**:** 1238-43.
